# Silica and Silica-Based Materials for Biotechnology, Polymer Composites, and Environmental Protection

**DOI:** 10.3390/ma15217703

**Published:** 2022-11-02

**Authors:** Jakub Zdarta, Teofil Jesionowski

**Affiliations:** Institute of Chemical Technology and Engineering, Faculty of Chemical Technology, Poznan University of Technology, Berdychowo 4, 60965 Poznan, Poland

**Keywords:** silica, biomaterials, silica-based materials, silica for biotechnology, polymer composites, environmental protection

Over recent years, silica and silica-based materials have become some of the most frequently used materials worldwide. This is due to the silica’s extraordinary stability and mechanical resistance, its neutral character for most molecules, and surface properties, such as well-developed surface area and the presence of numerous hydroxyl moieties. These features cause silica-based materials to be applied in various branches of everyday life, science, and industry. However, their use in biotechnology is still intensively expanding in materials chemistry. The extremely interesting and growing uses of silica for biotechnological applications include, among others, the adsorption of hazardous pollutants, catalysis, enzyme immobilization, drug-delivery systems, and the development of novel, eco-friendly solutions for multipurpose use. Further, the increasingly growing number of possibilities to design and develop novel synthesis approaches and use of advanced characterization techniques of silica and silica-based materials allows applying these materials, for instance, in sophisticated medical and catalytic solutions. Nevertheless, further scientific studies are still required as the development of novel silica-based materials is a driving force for the widespread application of the above-mentioned systems in various fields.

The scope of the presented Special Issue is extensive and multidisciplinary but still oriented towards silica and silica-based materials and their research. This Special Issue aims to include the latest findings on the synthesis, characterization, and application of silica and silicates in various areas. The published works cover several aspects related to the examination of the effect of various synthesis parameters on the properties of the final materials, as well as present different approaches for determining the properties of silica particles. Finally, the published articles present the application of silica-based materials in the pharmaceutical industry as a low-cost adsorbent and support material for enzyme co-immobilization.

Recently, numerous synthesis routes have been proposed to obtain silica materials with different morphologies and properties. Different approaches for synthesizing silica mesoporous silicas have been summarized in the review article presented by Jarmolinska et al. [1]. A special focus has been paid to Santa Barbara Amorphous (SBA) and Hybrid Mesoporous Materials (HMM) due to the widespread application of these silicas in the industry. Besides the general characterization of these materials and general procedures of their synthesis, data on silica modification with metal ions or/and organic functional groups have been presented to facilitate the practical application of these materials. In another study, Gan et al. [2] presented a novel approach for synthesizing a Cu-Al/SiO_2_ porous material to decompose NO. It has been reported that among all tested materials, the use of Cu-Al/SiO_2_ material with the template of cetyltrimethylammonium bromide resulted in 100% decomposition of NO at temperatures above 750 °C. A detailed study on the mechanism of denitrification showed that Cu (I) O should be considered as the active phase in the systems, whereas the redox reaction between Cu (II) O and Cu (I) O was considered the main mechanism of NO decomposition. In a study on the synthesis of silica materials, it is also important to clearly determine optimal process conditions and their effect on the properties of final products. With this aim, Tabisz et al. [3] used the classical Stöber silica synthesis protocol to determine the limits of simplification in the preparation route and size determination of silica nanoparticles. A direct relationship between the size of silica particles and concentrations of water and ammonia was presented. However, at optimized conditions, particles with diameters in the 15–400 nm range can be easily produced. It was also reported in this study that UV-Vis spectroscopy should be considered a simple and fast method of particle size determination with a level of error lower than 0.5 SD. Because the size of the particles is one of the most important parameters affecting the final properties of silica-based materials, the examination of effective and reproducible methods of particle size determination is of key importance. In this context, Al.-Khafaji et al. [4] tested four different methods, including transmission electron microscopy (TEM), small-angle X-ray scattering (SAXS), dynamic light scattering (DLS), and microfluidic resistive pulse sensing (MRPS) to characterize three inherently bimodal silica nanoparticles. Validation of the suitability of each technique is crucial, as most of the methods for synthesizing silica nanoparticles yield bimodal or even heterogeneous particle size distribution. The results showed that DLS and MRPS and SAXS have drawbacks, including problems with resolving bimodal particle size distribution, limitation to sizes of particles above 50 nm, as well as some mathematical difficulties for broad size distributions, respectively. Hence, it was concluded that the use of orthogonal techniques is required for the reliable description of the particle size distribution of silica nanoparticles.

Silica is also a main component of soil; hence, the processes that affect silica have a significant effect on soil properties. Among these, coastal accretion and erosion should be considered the most unavoidable, leading to modification and destabilization of soil. In a study by Eliaslankaran et al. [5], the geotechnical behavior of coast soil treated with lime, cement, and rice husk ash was followed to create a low-cost alternative mixture with environmentally friendly properties. The physical properties of the soil, followed by mechanical characteristics, were determined to examine the effect of different stabilizer/pozzolan ratios on the coastal soil. It was reported that the soil treated with lime and rice husk ash in the ratio of 1:2 showed, compared to the pristine soil, a significant improvement in shear stress followed by an increase in other strength parameters, such as cohesion and internal friction angle values. As previously mentioned, silicates are materials with various applications. These materials can also be used in the pharmaceutical industry as glidants in low concentrations, as well as in high concentrations as porous carriers and coating materials in oral solid drug-delivery systems. A study performed by Kominova et al. [6] demonstrated the characterization of commercially available magnesium aluminometasilicates (MAS) modified by microcrystalline cellulose as a potential glidant and/or binder. It was reported that the MAS concentration and pressure applied have a significant effect on MAS behavior. It was clearly presented that granulated, modified MAS exhibited an action-switching behavior. At low pressure, the interparticle interactions were reduced, leading to glidant action. By contrast, at higher pressure, these interactions were improved, leading to binder action. It was therefore summarized that by modifying conditions such as the pressure and modifier amount in the MAS, it was possible to prepare potential multifunctional excipients in pharmaceutical technology. Great sorption capacity, well-developed surface area, and the presence of numerous surface hydroxyl groups make it possible to apply silicas as support materials in enzyme immobilization. For instance, mesoporous SBA 15 silica was used as a carrier for the co-immobilization of glucose dehydrogenase and xylose dehydrogenase and further applied for the simultaneous production of gluconic acid and xylonic acid [7]. The effective enzymes’ co-immobilization was confirmed, and it was reported that a 1:5 GDH:XDH ratio was the most suitable for the efficient conversion of xylose and glucose, with a reaction yield of over 90%. Due to the protection of the silica support, a significant enhancement in the thermal, chemical, and storage stability of the co-immobilized enzymes was observed, followed by high relative activity over wider pH and temperature ranges compared to free enzymes. Finally, great reusability of the produced system was reported, as after five consecutive reaction steps, over 80% of the initial activity was retained, showing the great potential of silica in enzyme immobilization.

Articles collected in the Special Issue “*Silica and Silica-based Materials for Biotechnology, Polymer Composites and Environmental Protection*” clearly show the outstanding properties of silica and silica-based materials as well as indicate the enormous potential of these materials in various applications. Further, in any field of research, the collaboration between researchers and between researchers and industrial engineers is the key to innovation and the development of valuable solutions. Therefore, tracking the growing volumes of data and making the best possible use of them is highly important. Hence, we hope you enjoy reading this Special Issue and that it motivates you to find solutions to current problems and create new ideas for future and present research works. The editors would like to give special thanks to the authors of the manuscripts and the editorial team of *Materials* for the successful collaboration and efficient peer-review process.

## Data Availability

Not applicable.
